# Willingness to Pay for HIV Prevention Commodities Among Key Population Groups in Nigeria

**DOI:** 10.9745/GHSP-D-21-00303

**Published:** 2022-10-31

**Authors:** Olawale Durosinmi-Etti, Emmanuel Kelechi Nwala, Funke Oki, Akudo Ikpeazu, Emmanuel Godwin, Paul Umoh, Arome Shaibu, Alex Ogundipe, Abiye Kalaiwo

**Affiliations:** a JSI Research and Training Institute Inc, Abuja, Nigeria.; b National Agency for the Control of AIDS, Abuja, Nigeria.; c National AIDS and STIs Control Program, Federal Ministry of Health, Abuja, Nigeria.; d Heartland Alliance Nigeria, Abuja, Nigeria.; e U.S. Agency for International Development Nigeria, Abuja, Nigeria.

## Abstract

**Introduction:**

Key population (KP) groups, such as female sex workers and men who have sex with men, in Nigeria rely on free HIV prevention commodities, including pre-exposure prophylaxis (PrEP) and HIV self-testing (HIVST) kits, provided through foreign aid. We investigated the willingness of KP groups to use and pay for HIV prevention commodities to support improved sustainable HIV prevention programming.

**Methods:**

In 2020, we conducted a cross-sectional survey in 3 states with KP groups. The survey covered sociodemographic characteristics and willingness to use and pay for PrEP, HIVST, and condoms, and we used a bidding game iteration process to collect data on factors that influence willingness to use and pay for the commodities. We performed bivariate and multivariable regression analyses to explore factors that may determine willingness to pay and the maximum amount willing to pay.

**Results:**

Of the participants surveyed, 73% were willing to pay for PrEP services, 81% were willing to pay for HIVST, and 87% were willing to pay for condoms. Willingness to pay varied between the commodities and was associated with, among other variables: age, KP group, marital status, level of education, employment status, place of residence, average monthly income, and familiarity with the commodity in question.

**Conclusion:**

We demonstrate that KP groups are willing to pay for HIV prevention commodities, but there is a need to bridge the gap between the maximum amount they are willing to pay and retail prices. If prices are reduced, the willingness to pay may result in high consumption and positive returns for the private sector.

## Abstract (precis)

Exploring the willingness of certain population segments to pay for HIV commodities is the first step in developing strategies to maximize the use of free or subsidized products, increase access to products for key segments of the population, and ensure efficiency and sustainability of HIV prevention programming.

## Teaser

**Key Findings:**

- Overdependence on donor funding to provide HIV commodities through the public sector in Nigeria is not sustainable and has created gaps in HIV prevention programming.
- Nigeria adopted an approach to look across public and private sectors to increase access to HIV prevention commodities, such as pre-exposure prophylaxis, HIV self-testing, and condoms, thereby reducing the risk of acquiring HIV infections, particularly among key population (KP) groups at high risk of infection, including female sex workers (FSWs) and men who have sex with men (MSM) who rely on free commodities.
- Exploring willingness to pay for commodities among KP groups helped elucidate effective strategies for HIV prevention programming while maximizing limited resources. It will help ensure better targeting of the key populations with HIV prevention commodities.

**Key Implications:**

- Stakeholders must work together to negotiate the pricing for HIV prevention commodities, and the government should consider providing tax waivers for imported commodities to support the expansion of access through the private sector.
- KP groups should be encouraged to enroll in health insurance schemes and the Basic Health Care Provision Fund to ensure equitable access to HIV prevention services.

## BACKGROUND

In Nigeria, female sex workers (FSWs), men who have sex with men (MSM), and people who inject drugs, which we refer to as key population (KP) groups in this article, constitute about 3.4% of the country’s total population and contribute about 32% to new HIV infections.^1^ Amidst high HIV prevalence, the country adopted a combination prevention strategy, including the use of pre-exposure prophylaxis (PrEP), HIV self-testing (HIVST), and condoms, to ensure reduced risk of HIV infection among KP groups. A continuing increase in sexual risk behaviors among the sexually active population suggests the need for an expanded effort to ensure sustainable programming for these HIV prevention commodities.^2^

PrEP and HIVST can help prevent new infections and increase the uptake of HIV testing, respectively, and may support countries to achieve the 95–95–95 targets to end the AIDS epidemic by 2030.^3^^,^^4^ HIVST has the potential benefit of reaching untested individuals—including KP groups who infrequently engage with the health care system—thereby drastically reducing the number of undiagnosed HIV cases. PrEP significantly reduces the risk of contracting HIV through sex by about 99% and through injection drug use by 74% when taken daily.^5^ However, reports have also highlighted the importance of adherence to dosing for effective results.^6^^,^^7^

Since Nigeria adopted condom use as one of its HIV priority prevention pillars, use has increased to the point that 74% of the sexually active population use condoms.^1^^,^^8^^,^^9^ Among KP groups and their clients, condom use is currently estimated at 91%, 80%, and 83% for FSWs, MSM, and people who inject drugs, respectively.^2^^,^^10^ However, a majority of these KP groups rely heavily on free condoms provided through foreign aid.

As of 2020, of the Nigerian government’s total investment in the HIV program, 67% was used for prevention, 3% for testing services, and the remaining 30% on treatment services. Almost all (98%) of commodities’ expenditure for prevention is funded by donor investments with the U.S. President’s Emergency Plan for AIDS Relief contributing 82% and the Global Fund contributing 16%. Only 2% of commodities investments received domestic funds.

The nearly total reliance on donor funding for commodity provisions is not sustainable nor does it provide opportunities for the strengths of the private, commercial, and social marketing sectors to be harnessed. To that end, in 2015, Nigeria adopted a total market approach to ensure sustainable access to condoms, HIVST, and PrEP. A total market approach is a process to develop strategies that increase access to priority health products for all segments of the population. In other words, the approach helps expand the market for HIV commodities by maximizing the use of free or subsidized products, reducing inefficiencies and overlaps, and creating room for the private sector to increase its provision of health commodities under government stewardship.

Nigeria adopted a total market approach to HIV prevention to ensure sustainable PrEP, HIVST, and condom programming and that the comparative advantages of the private, commercial, and social marketing sectors are better harnessed.

One method of market segmentation, a crucial first step in the total market approach process, involves exploring the willingness to pay among key segments of the population. Currently, there is little contextual evidence available in Nigeria about the willingness of FSW and MSM to use and pay for HIV prevention commodities. Therefore, we investigated the willingness to pay for and use HIV prevention commodities among FSW and MSM in Nigeria as a way to support improved sustainable HIV prevention commodity investments. Specifically, we aimed to determine the proportion of KP groups who are willing to pay for HIV prevention commodities, the median amount the KP groups are willing to pay for each commodity, factors that affected willingness to pay for each commodity, and recommended strategies for improved and sustainable HIV prevention programming in Nigeria.

## METHODS

### Study Design and Setting

We used a cross-sectional survey design with a total of 1,169 participants, including 824 FSWs and 345 MSM in Akwa Ibom, Cross River, and Lagos states, which are priority states for the United States Agency for International Development-sponsored Total Market Approach to HIV Prevention Programming project, funded through a sub-award from Heartland Alliance LTD/GTE, Nigeria. The project supports the government of Nigeria and implementing partners to enhance the sustainability of HIV prevention services including condoms, PrEP, HIVST, and lubricants.

### Participants

The study population consisted of FSWs and MSM in Akwa Ibom, Cross River, and Lagos states. We created a sampling frame of the KP groups based on previous KP groups’ characterization in 2015 by the Society for Family Health^11^ and information from the KP groups’ networks. Next, we used a stratified sampling technique to categorize the KP groups according to typology (FSWs versus MSM). For each state, a proportionate sampling technique was used to arrive at the minimum sample size. Finally, the respondents were selected using a simple random sample without replacement until the minimum sample size was reached.

### Sample Size Calculation

We used Slovin’s formula to estimate the sample size at a 95% confidence interval (CI) and a power of 5% error tolerated.

$$\text{Sample size (n) = }\frac{N}{1+Ne^{2}}$$

where n is the required sample size, N is the population size, and e is the margin of error (5%).

Only participants aged 18 years and older and who gave consent participated in the study.

### Data Collection

In August 2020, we deployed a pretested, interviewer-administered questionnaire in an open data kit application on mobile smartphones. We recruited the participants through their networks led by key influencers. The questionnaire format on the open data kit used skip patterns to reduce bias/error and limit missing values. Questionnaires were devoid of personal identifiers to ensure confidentiality. We collected data in English, except where otherwise indicated by the respondent. KP groups' sociodemographic characteristics; willingness to pay for PrEP, HIVSTs, and condoms; and factors that influence KP groups’ willingness to pay for the prevention commodities were collected. Unlike discrete choice experiment that lacks direct valuation questions, the contingent valuation method has the advantage of being the best approach to reducing misunderstanding in face-to-face contact.^12^

### Estimation of Willingness to Pay Amount: The Bidding Game Iteration Process

Among all the contingent market valuation techniques, the bidding game closely depicts the usual price-taking structure in Nigeria and has been widely used.^13^^–^^17^ The bidding game iteration process allows respondents to truncate up and down the price range before finally converging at the maximum amount they are willing to pay for a commodity. There were different starting points for PrEP, HIVST, and condoms. The interviewer introduced PrEP, HIVST, and condoms to each participant and then asked if they had ever purchased PrEP, HIVST, and condoms, before proceeding with a set of questions about the mid-range-priced HIVST and generic brand of oral tenofovir/emtricitabine used for PrEP in the country. Participants were asked to indicate if they were willing to pay the current retail market price for PrEP, HIVST, and condoms in Nigeria. A higher bidding amount for PrEP, HIVST, and condoms was asked based on the responses. Table 1 describes the different price starting points for the commodities.

**TABLE 1.** Starting and Ending Points for the Bidding Game Iteration Process to Estimate the Amount Survey Respondents Were Willing to Pay^a^ for HIV Commodities in Nigeria

	PrEP, Nigerian Naira (US$)	HIVST, Nigerian Naira (US$)	Condoms, Nigerian Naira (US$)
Starting point	2,000 (5.30)	1,000 (2.60)	150 (0.40)
	2,500 (6.60)	1,500 (4.00)	225 (0.60)
	3,000 (7.90)	2,000 (5.30)	300 (0.90)
	3,500 (9.20)	2,500 (6.60)	375 (1.00)
	4,000 (10.50)	3,000 (7.90)	450 (1.18)
	4,500 (11.80)		

Abbreviations: HIVST, HIV self-testing; PrEP, pre-exposure prophylaxis.

a Nigerian Naira 380=US$1.

### Data Management and Analysis

Data were uploaded into the Kobo Toolbox server daily and exported into an Excel-based spreadsheet. We conducted data cleaning daily, checking for consistency and completeness before the study team left the study site and before importing the data into statistical software. We used SPSS (Version 20) to determine descriptive and inferential statistics. Bivariate and multivariate analyses were performed to determine factors that may determine willingness to pay and the maximum amount willing to pay for PrEP, HIVST, and condoms. Bivariate and multivariable analysis was conducted between dependent variables (willingness to pay for and maximum amount willingness to pay) and independent variables (age, KP group, marital status, level of education, employment status, place of residence, average monthly income, awareness, knowledge, satisfaction, willingness to receive future PrEP/HIVST messages, and previous experience using PrEP/HIVST).

### Ethical Approval

The research protocol was approved by the National Health Research Ethics Committee of the Federal and State Ministries of Health, as well as by the Health Research (NHREC/01/01/2007-28/08/2020) and the Ethics Committee of John Snow, Inc. Institutional Review Board. Consent was sought from each of the study participants and duly documented.

## RESULTS

### Sociodemographic Characteristics

The majority of the participants were single (84% of FSWs, 98% of MSM), aged 18–28 years (64% of FSWs, 89% of MSM), completed secondary education (56% of FSWs, 44% of MSM), and self-employed (56% of FSWs, 40% of MSM). For participants who were employed, the total average monthly income was less than the Nigerian national minimum wage (mean: (Nigerian Naira [N]27,661) [US$59] and median: N15,000 [US$39]). The earning power was low; only about 28% of MSM and 26% of FSW groups earned above the national minimum wage (Table 2).

**TABLE 2.** Sociodemographic Characteristics of Survey Respondents in 3 States in Nigeria

	FSW, No. (%)	MSM, No. (%)
Sample size, N=1,169	824 (70.5)	345 (29.5)
Akwa Ibom, n=368	225 (61.1)	143 (38.9)
Cross River, n=385	286 (74.3)	99 (25.7)
Lagos, n=416	313 (75.2)	103 (24.8)
Age		
18–28 years	526 (63.8)	308 (89.3)
29–38 years	229 (27.8)	34 (9.9)
Older than 38 years	69 (8.4)	3 (0.9)
Marital status		
Married	38 (4.6)	8 (2.3)
Divorced/separated	75 (9.1)	0 (0)
Single	689 (83.6)	337 (97.7)
Widowed	22 (2.7)	0 (0)
Sexual orientation		
Bisexual	421 (51.1)	145 (42)
Homosexual	43 (5.2)	191 (55.5)
No response	47 (5.7)	9 (2.6)
Other	313 (38)	0 (0)
Level of education		
Primary not completed	60 (7.3)	0 (0)
Primary completed	94 (11.4)	2 (0.6)
Junior secondary completed	115 (14)	1 (0.3)
Senior secondary completed	462 (56.1)	150 (43.5)
Advanced level	10 (1.6)	6 (1.7)
Undergraduate	78 (9.5)	177 (51.3)
Postgraduate	5 (0.6)	9 (2.6)
Occupation		
Government employed	3 (0.4)	9 (2.6)
Private sector	18 (2.2)	30 (8.7)
Self-employed	465 (56.4)	138 (40)
Student	52 (6.3)	137 (39.7)
Unemployed	210 (25.8)	57 (16.5)
Others (e.g., retired)	76 (9.2)	4 (1.4)
Place of residence		
Rural	304 (36.9)	77 (23.3)
Urban	520 (63.1)	268 (77.7)
Average monthly income^a^		
<N30,000 (<US$79)	374 (74.1)	149 (71.6)
≥N30,000 (>US$79)	131 (25.9)	59 (28.4)

Abbreviations: FSW, female sex worker; KP, key population; MSM, men who have sex with men.

a Nigerian Naira (N)380=US$1.

### Previous Experience of Use of PrEP Services

The history of previous use of PrEP among the participants was generally low, though higher in Akwa Ibom (27%) compared to Lagos (24%) and Cross River (9%). Considering variations in previous use of PrEP services by KP typology, PrEP use was higher among the MSM (23%) than the FSWs (19%). In Akwa Ibom, the FSW group reported higher previous use of PrEP than the MSM while in Cross River and Lagos PrEP use was higher among the MSM group than FSWs. In Lagos, MSM (34%) reported higher use than FSWs (20.4%).

Factors that discouraged the previous use of PrEP were elicited including having to use PrEP daily (41% of FSWs and 51% of MSM). More participants in Akwa Ibom and Lagos were negatively influenced by having to use PrEP daily. Other factors included the need to co-use condoms while on PrEP (16% of FSW and 17% of MSM) and consideration of certain sex partners as “special”—that is someone they can trust (17% of FSW and 16% of MSM). In contrast, the low cost of PrEP because user fees were eliminated promoted previous use of the commodity (40% of FSW and 37% of MSM). Most participants currently access PrEP free of charge.

### Willingness to Use and Pay for PrEP

A total of 73% of the participants were willing to pay for PrEP services (77% of FSWs and 65% of MSM). More participants in Lagos and Cross River indicated a willingness to pay (76% and 76%, respectively) compared to participants in Akwa Ibom (67%). The maximum amount participants were willing to pay varied between the KP typologies and was higher among the MSM group. About 17% of all the participants (14% of FSWs and 23% of MSM) were willing to pay a maximum of N3,500 (US$9.20) (the market price of PrEP) and above. The findings showed that about 36.1% of KP groups were willing to pay if PrEP cost N2,500–N3,400 (US$6.60–US$9.00). However, understanding the previous payment history for PrEP indicated minimal evidence of informal payments for PrEP services among KP groups with a history of previous use (0.5% for MSM and 2.3% for FSWs), suggesting that a vast majority previously received PrEP services free of charge.

The maximum amount participants were willing to pay for PrEP was higher among the MSM group.

### Factors That Affected Willingness to Pay for PrEP Services

We explored factors that affected the dependent variable (willingness to pay for PrEP) and independent variables (age, KP group, marital status, level of education, employment status, place of residence, average monthly income, awareness, knowledge, satisfaction, willingness to receive future PrEP messages, and previous experience using PrEP). At a 95% CI, willingness to pay for PrEP correlated with the KP group, occupation, and willingness to receive future PrEP messaging (*P*<.05; 95% CI). Nonetheless, marital status, education, place of residence, income, PrEP awareness, and previous use of PrEP did not show any relationship with willingness to pay for PrEP (*P*>.05; 95% CI). Multivariable regression was used to explore the relationship between sociodemographic and socioeconomic factors and the KP groups’ willingness to pay for PrEP. These factors did not show any association with willingness to pay for PrEP (Table 3).

**TABLE 3.** Factors That Affected Willingness to Pay for PrEP Among KPs in 3 States in Nigeria

	Bivariate		Multivariate	
	*P* Value	95% CI	Odds Ratio	95% CI
Age younger than 28 years	.737	−0.069, 0.047	−0.189	0.417, 1.643
MSM	.000^a^	−0.189, 0.071	−0.760	0.245, 0.893^a^
Married	.817	−0.050, 0.068	−0.497	0.121, 3.054
Secondary level of education and higher	.620	−0.040, 0.073	0.528	0.769, 3.740
Employed	.003^a^	−0.144, 0.026	−0.475	0.345, 1.122
Urban place of residence	.780	−0.051, 0.065	−0.470	0.344, 1.133
Average monthly income^b^ of <N30,000 (<US$79)	.391	−0.037, 0.103	0.292	0.704, 2.546
Had ever heard of PrEP	.158	−0.015, 0.098	0.098	0.399, 3.049
Had some knowledge of PrEP	.105	−0.008, 106	–	–
Satisfied with PrEP message quality	.342	−0.123, 0.044	−0.18	0.454, 1.573
Willing to receive PrEP messages in future	.000^a^	0.164, 0.295	0.979	0.721, 9.821
Had ever used PrEP	.102	−0.008, 0.102	0.438	0.851, 2.824

Abbreviations: CI, confidence interval; FSW, female sex worker; KP, key population; MSM, men who have sex with men; PrEP, pre-exposure prophylaxis.

a Statistically significant.

b Nigerian Naira (N)380=US$1.

### Factors That Affected Maximum Amount Willing to Pay for PrEP Services

We explored factors that affected the dependent variable (maximum amount willing to pay) and independent variables (age, KP group, marital status, level of education, employment status, place of residence, average monthly income, awareness, knowledge, satisfaction, willingness to receive future PrEP messages, and previous experience using PrEP). Both bivariate and multivariable regression analyses found that the maximum amount the KP groups were willing to pay was associated with participants’ awareness of PrEP services. Participants who had previous knowledge about PrEP expressed a higher willingness to pay N3,500 (US$9.20) for PrEP services (odds ratio [OR]∼1.854; 95% CI=0.765, 53.335). We conducted a bivariate analysis to determine the association between KP typology and knowledge of PrEP on the maximum amount willing to pay, and we found an association. However, multivariate analysis did not show any associations between any of these independent variables (Table 4).

**TABLE 4.** Factors That Affected Maximum Willingness to Pay for PrEP Among KPs in 3 States in Nigeria

	Bivariate		Multivariate	
	*P*Value	95% CI	Odds Ratio	95% CI
Age younger than 28 years	.064	−0.123, 0.070	0.018	0.464, 2.232
MSM	.001^a^	0.042, 0.183	0.515	0.769, 3.642
Married	.790	−0.082, 0.057	−0.243	0.188, 3.266
Secondary level of education and higher	.467	−0.045, 0.087	−0.134	0.346, 2.207
Employed	.604	−0.049, 0.083	0.422	0.71, 3.255
Rural residence	.067	−0.130, 0.008	0.902	0.209, 0.790
Average monthly income^b^ <N30,000 (<US$79)	.151	−0.029, 0.153	0.467	0.782, 3.255
Had ever heard of PrEP	.033^a^	0.008, 0.139	1.854	0.765, 53.335^a^
Had some knowledge of PrEP	.022^a^	0.011, 0.149	0	0
Satisfied with PrEP message quality	.500	−0.060, 0.121	0.055	0.503, 2.220
Willing to receive PrEP messages in future	.359	−0.114, 0.039	−1.399	0.042, 1.560
Had ever used PrEP	.178	−0.018, 0.117	−0.290	0.370, 1.514

Abbreviations: CI, confidence interval; FSW, female sex worker; KP, key population; MSM, men who have sex with men; PrEP, pre-exposure prophylaxis.

a Statistically significant.

b Nigerian Naira (N)380=US$1.

### Previous Experience of Use of HIVST Kits

The previous experience of use of HIVST kits was higher among the MSM (21%) group compared to FSWs (17%). Geographic differences exist in the history of previous use of HIVST services; however, this was higher in Akwa Ibom (20%) compared to Cross River (19%) and Lagos (14%). Also, findings showed typological differences across the states. For example, 27% of MSM had experience using HIVST compared to 10% of FSWs in Lagos. Whereas in Cross River, the proportion of FSWs was higher than MSM (12%). However, the trend is different, as almost the same proportions of the KP groups reported previous experience with the use of HIVST kits.

### Willingness to Pay for HIVST Kits

About 81% of participants were willing to pay for HIVST services (84% of FSWs and 74% MSM). A total of 32% (31% of FSWs and 36% of MSM) were willing to pay a maximum amount of N1,600 (US$4.20) (the market price of an HIVST kit) or higher. The average (mean) willingness to pay amount was N1,420 (US$3.70), while the median was N1,000 (US$2.60). There were geographic variations in willingness to pay. However, the analysis found that an additional 42% of KP groups were willing to pay to access HIVST kits if they cost N500–N1,500 (US$1.30–US$4.00) each.

About 81% of participants were willing to pay for HIVST services.

### Factors That Affected Willingness to Pay for HIVST Kits

Bivariate and multivariable analysis was conducted between willingness to pay for HIVST (dependent variable) and age, KP group, marital status, level of education, employment status, place of residence, average monthly income, awareness, knowledge, satisfaction, willingness to receive future HIVST messages, and previous experience using HIVST (independent variables). The level of education, awareness, and satisfaction with the quality of HIVST messages exerted an association with the willingness to pay for HIVST kits (95% CI). This implies that participants with secondary education and above were more willing to pay compared to those who did not have secondary education. Also, participants who were aware of HIVST before the study were more likely to pay for the kit, and the more satisfied the respondents were, the more likely they would be willing to pay for the kits. Despite bivariate analysis yielding an association between willingness to pay for HIVST kits, if they ever received messaging and were willing to participate in future HIVST messaging, the multivariable analysis did not find any association between willingness to pay and these independent variables (Table 5).

**TABLE 5.** Factors That Affected Willingness to Pay for HIVST Kits Among KPs in 3 States in Nigeria

Independent Variable	Bivariate		Multivariate	
	*P* Value	95% CI	Odds Ratio	95% Cl
Age younger than 28 years	.532	−0.075, 0.038	−0.505	−1.516, 0.516
MSM	.000^a^	−0.177, −0.057^a^	−1.797	−2.962, −1.029^a^
Married	.943	−0.051, 0.061	−0.215	−19.799, 1.571
Secondary level of education and higher	.227	−024, 0.096	0.916	−0.218, 1.881^a^
Employed	.773	−0.062, 0.050	−0.139	−1.998, 0.829
Rural residence	.999	−0.061, 0.054	−0.342	−1.1288, 0.383
Monthly income^b^ <N30,000 (<US$79)	.537	−0.053, 0.091	0.489	−0.411, 1.917
Had ever heard of HIVST	.011^a^	0.016, 0.128^a^	1.296	0.158, 2.506^a^
Had ever received messaging about HIVST	.001^a^	0.040, 0.151^a^	0	(0)
Satisfied with HIVST message quality	.073	−0.008, 0.180	0.928	0.099, 1.956^a^
Willing to receive HIVST messages in future	.000^a^	0.147, 0.301^a^	−0.677	−20.562, 1.536
Had ever used HIVST	.092	0.000, 0.103	−0.604	−1.556, 0.172

Abbreviations: CI, confidence interval; FSW, female sex worker; HIVST, HIV self-testing; KP, key population; MSM, men who have sex with men.

a Statistically significant.

b Nigerian Naira (N)380=US$1.

### Factors That Affected Maximum Amount Willing to Pay for HIVST Services

Bivariate and multivariable analyses explored the association between maximum amount willing to pay and age, KP group, marital status, level of education, employment status, place of residence, average monthly income, awareness, knowledge, satisfaction, willingness to receive future HIVST messages, and previous experience using HIVST (independent factors). Findings indicated that employment status (OR∼0.728; 95% CI=1.116, 3.842) and monthly income (OR∼0.988; 95% CI=1.465, 4.928) have a positive association with the maximum amount they were willing to (Table 6).

**TABLE 6.** Factors That Affected Maximum Willingness to Pay Amount for HIVST Kits Among KPs in 3 States in Nigeria

	Bivariate		Multivariate	
	*P* Value	95% CI	Odds Ratio	95% CI
Age				
Younger than 28 years	.465	−0.076, 0.037	0.273	0.701, 2.460
KP group				
MSM	.077	−0.010, 0.109	0.372	0.781, 2.694
Marital status				
Married	.931	0.063, 0.050	−0.695	0.150, 1.661
Secondary and above	.871	−0.055, 0.062	−0.041	0.467, 1.972
Employment status				
Employed	.956	−0.058, 0.060	0.728	1.116, 3.842^a^
Place of residence				
Rural	.229	−0.099, 0.023	−0.175	0.486, 1.450
Monthly income^b^				
<N30,000 (<US$79)	.000^a^	0.093, 0.245^a^	0.988	1.465, 4.928^a^
Ever heard of HIVST				
Yes	.965	−0.058, 0.057	0.390	0.567, 3.849
Ever received messaging about HIVST				
Yes	.644	−0. 049, 0.071	0	0
Satisfaction with the quality of HIVST messages				
Yes	.347	−0.135, 0.049	−0.376	0.379, 1.244
Willing to receive HIVST messages in future				
Yes	.036^a^	0.011, 0.112^a^	0.193	0.273, 5.388
Ever used HIVST				
Yes	.064	−0.108, 0.001	−0.565	0.322, 1.003^a^

Abbreviations: CI, confidence interval; FSW, female sex worker; HIVST, HIV self-testing; KP, key population; MSM, men who have sex with men.

a Statistically significant.

b Nigerian Naira (N)380=US$1.

### Willingness to Pay for Condoms

About 87% of participants were willing to pay for condoms. About 38% of the participants were willing to pay N100–N150 (US$0.30–US$0.40) per condom. Our findings show that the higher the price of condoms, the lower the demand. The MSM group indicated a higher willingness to pay for condoms than the FSWs.

### Factors That Affected Willingness to Pay for Condoms

Associations between willingness to pay for condoms (the dependent variable) and age, KP group, marital status, level of education, employment status, place of residence, sexual activity use of condoms, monthly income, and other factors (independent variables) were explored. Our findings revealed that willingness to pay for condoms increased with education above the secondary level (OR∼0.863; 95% CI=0.893, 6.288). Willingness to purchase condoms was associated with KP typology. In other words, MSM were more willing to pay for condoms than FSWs (OR∼4.342; 95% CI=2.932, 2014.322). The more recent the sex act, the higher the willingness to pay for condoms (OR∼4.026; 95% CI=5.120, 613.506). Similarly, participants were likely to pay for subsequent condom use if they purchased it for the last sex act (OR∼1.256; 95% CI=1.283, 9.604) (Table 7).

**TABLE 7.** Factors That Affected Willingness to Pay for Condoms Among KPs in 3 States in Nigeria

Independent Variable	Bivariate		Multivariate	
	*P* Value	95% CI	Odds Ratio	95% CI
Age younger than 28 years	.947	−0.112, 0.092	0.185	0.406, 3.565
MSM	.008^a^	0.059, 0.205^a^	4.342	2.932, 2014.322^a^
Married	.581	−0.085, 0.073	−18.751	−20.083, −13.633
Secondary level of education and higher	.183	−0.042, 0.175	0.863^a^	0.893, 6.288^a^
Employed	.438	−0.061, 0.134	−0.368	0.241, 1.989
Urban residence	.098	−0.002, 0.208	−0.646	0.192, 1.431
Monthly income^b^ <N30,000 (<US$79)	.793	−0.155, 0.113	−0.722	0.138, 1.705
Had sexual activity in the last 3 months	.017^a^	−0.005, 0.256^a^	4.026	5.120, 613.506^a^
Used condom during last sexual activity	.267	−0.047, 0.172	0	0
Purchased condom for last sex	.238	−0.062, 0.163	1.256	1.283, 9.604^a^
Believed protection from STIs can make me use condoms	.973	−0.093, 0.114	0.653	0.068, 54.266
Believed protection from unintended pregnancy can make me use condoms	.116	−0.158, 0.000	−1.403	0.009, 6.499

Abbreviations: CI, confidence interval; FSW, female sex worker; KP, key population; MSM, men who have sex with men; STI, sexually transmitted infection.

a Statistically significant.

b Nigerian Naira (N)380=US$1.

### Factors That Affected the Maximum Amount That KP Groups Are Willing to Pay for Condoms

The maximum amount that participants were willing to pay for condoms was associated with age, typology, employment status, place of residence, and previous purchase of condoms in the last sex act. Participants aged older than 28 years were more willing to pay N350 (US$0.90) or higher for commercial condoms (OR∼1.002; 95% CI=0.207, 2.020). Both bivariate and multivariate analyses found an association between KP typology and the maximum amount they were willing to pay—FSWs have more tendency to pay N350 (US$0.90) for commercial condoms compared to MSM groups (OR∼2.271; 95% CI=0.927, 4.339). Also, we found an association between the maximum amount willing to pay and employment status (Table 8).

**TABLE 8.** Factors That Affected Maximum Willingness to Pay Amount for Condoms Among KPs in 3 States in Nigeria

	Bivariate		Multivariate	
	*P* Value	95% CI	Odds Ratio	95% CI
Age younger than 28 years	.071	−0.016, 0.198	1.002	0.207, 2.020^a^
MSM	.002^a^	0.057, 0.269^a^	2.271	0.927, 4.339^a^
Married	.738	−0.088, 0.102	0.686	−1.861, 20.709
Level of education				
Secondary and above	.102	−0.008, 0.172	−0.032	−1.143, 1.120
Employed	.647	−0.073, 0.134	0.904	0.160, 1.890^a^
Urban residence	.325	−0.148, 0.053	−1.529	−2.999, −0.599^a^
Monthly income^b^ <N30,000 (<US$79)	.368	−0.178, 0.063	−0.969	−2.577, 0.157
Had sexual activity in the last 3 months	.005	−0.256, −0.032^a^	−0.650	−2.820, 1.363
Used condom during last sexual activity	.959	−0.107, 0.097	0	0
Purchased condom for last sex	.169	−0.029, 0.179	1.019	0.183, 2.344^a^
Believed protection from STIs can make me use condoms	.391	0.055, 0.125	0.139	−2.661, 20.182
Believed protection from unintended pregnancy can make me use condoms	.470	−0.146, 0.071	−0.504	−2.772, 1.417

Abbreviations: CI, confidence interval; FSW, female sex worker; KP, key population; MSM, men who have sex with men; STI, sexually transmitted infection.

a Statistically significant.

b Nigerian Naira (N)380=US$1.

## DISCUSSION

Most participants in the study earned below the Nigerian minimum wage, which could be a reflection of the poor state of the economy.^15^^,^^17^ This low earning capacity may affect participants’ willingness to pay to access HIV prevention services. Differences in earning further highlight the importance of segmenting populations based on willingness and ability to pay for HIV prevention services. This will ensure that free services target low-income individuals, eliminating those in the upper wealth quantile who can then maximize the subsidized and full-priced commercial sector. However, age may be co-confounded by unemployment thereby limiting access to essential HIV prevention commodities.

Differences in earning highlight the importance of segmenting populations based on willingness and ability to pay for HIV prevention services.

### PrEP

The amount the participants were willing to pay for PrEP was lower than the retail market price. More so, less than one-quarter (17%) of the participants indicated a willingness to pay the market price for PrEP services, estimated to be N3,500 (US$9.20). Our findings suggest that if there are brands of antiretroviral medicines for PrEP available within a price range of N2,500–N3,400 (US$6.60–US$8.90), 36% of KP groups will be willing to pay for PrEP services. Interestingly, recent retail market audits have shown different brands of PrEP that are within this price range, although the majority of these brands do not have National Agency for Food and Drug Administration and Control authorization. If the agency authorizes other available brands selling for N2,500–N3,400 (US$6.60–US$8.90), more participants may purchase PrEP. That will also ensure competitive choice and may lead to pricing reductions for PrEP.

Factors, such as place of residence and awareness, that influenced the maximum amount that participants were willing to pay for PrEP must be considered when designing an enabling market environment for increased access and use of PrEP services.^17^^–^^20^ Because this study revealed a relationship between previous awareness about PrEP and the maximum amount willing to pay, scaling access to PrEP services may benefit from social marketing approaches that allow commensurate consumer awareness campaign strategies, as reported in other studies.^17^^–^^20^

### HIVST

In this study, we found that HIVST was gaining attention, although some of the participants do not worry about any method of inquiry for HIV status. That is in line with the findings of other studies.^21^ This finding is encouraging but requires careful consideration of the factors that influenced previous use of HIVST, including convenience, the accuracy of results, and ease of instruction.^22^^–^^26^

The study elicited an average willingness to pay an amount for HIVST that is lower than the retail market price. However, total market approach efforts must continue to incorporate pricing into the market development intervention packages while engaging private sector players and designing social and behavior change packages to ensure sustainable access to HIVST kits in Nigeria. Predicting factors influencing willingness to pay and use HIVST, such as level of education, awareness, and satisfaction with the quality of HIVST messages, should not be underestimated. We did not investigate the impact of educational status on the usability of HIVST kits by the respondents. Nonetheless, strategies to gather tailored feedback on the usability of the kits from the test counselors and key market players are crucial.^27^

TMA efforts must continue to incorporate pricing into the market development intervention packages while engaging the private sector players and designing social and behavior change packages to ensure sustainable access to HIVST kits in Nigeria.

Employment status and monthly income positively influenced the maximum amount participants were willing to pay for HIVST kits. Participants who were employed were more likely to pay a maximum of N1,600 (US$4.20) or more. The maximum amount participants were willing to pay increases as income increases. However, despite a relatively small proportion of the KP groups to the total Nigerian population, the participants merit closer attention due to increased sexual health risks. We reported that about 32% of the participants expressed willingness to purchase HIVST kits at N1,600 (US$4.20) or more (retail price). This implies that effective market development strategies such as negotiating competitive pricing with the private sector, cross-subsidization, and social marketing can support an additional 42% to pay N500–N1500 (US$1.30–US$4.00) to have quality access to HIVST kits.

### Condoms

When we explored sexual risk behaviors among the participants, FSWs reported more frequent sexual activity than the MSM group. That did not seem surprising due to the commercial nature of FSWs' sexual activity. However, it is concerning that about 17% of last sex acts were reported to be without a condom. Bridging this gap may require sustained condom awareness campaigns that resonate with purchasing condoms, including social marketing.^28^^,^^29^ There seems to be a measurable success in the social market and commercial windows to expanding condom access and use in Nigeria, as evidenced by the recent condom market trend analysis conducted by the Total Market Approach to HIV Prevention Programming project in 2020. Most participants purchased condoms for recent sexual activities with typological disparities.

The findings illustrated the dynamism in the retail market for condoms in Nigeria. Even with disparities among the KP groups, willingness to pay for condoms peaked at N100–N150 (US$0.30–US$0.40) with market flexibilities. However, the willingness to pay at each of the price schedules is not attributable to the brand of condoms. Therefore, further willingness to pay for condoms must consider the brands to enable specificity and sensitivity while increasing access to condoms.

The willingness to pay a maximum of N350 (US$0.90) for a pack of 3 condoms was higher among older individuals in KP groups. It may be crucial to use older individuals in KP groups as condom ambassadors for sensitizing others on priced condom use in Nigeria. A strong association exists between KP typology and the maximum amount willing to pay. FSWs are likely to pay N350 (US$0.90) or more for priced condoms. The more the participants are employed, the more they purchase priced condoms.

### Next Steps

Our findings imply that to reduce overdependence on public funds to provide PrEP, HIVST, and condom services it is necessary to segment the participants based on free, subsidized, and fully commercialized access and ensure they are targeted based on the willingness and ability to pay. However, a multisectoral effort is required to effectively negotiate competitive pricing for these commodities. The government should consider providing tax waivers for imported commodities to support private sector involvement in expanding access to PrEP, HIVST, and condoms. KP groups should also be encouraged to enroll in health insurance schemes and Basic Health Care Provision Fund to ensure equitable access to HIV prevention services. Continuous advocacy and engagement with KP groups may increase awareness and encourage those who can pay for HIV prevention services, which in turn will help ensure financial risk protection for low-income and unemployed individuals.

A multisectoral effort is required to effectively negotiate competitive pricing for PrEP, HIVST, and condom services.

### Limitations

Although the 3 states surveyed in the study have some of the highest burdens of HIV infection, increasing the geographic scope of this research is necessary to ensure the representativeness of the socioeconomic characteristics between states in Nigeria and allow for a maximum generalization of findings. Additionally, this study did not elicit preferences for brands. A further study must consider eliciting preferences for brands of PrEP, HIVST, and condoms to inform supply-side strategy designs in Nigeria. We did not offer the actual goods (PrEP, HIVST, and condoms) to the participants who are the prospective buyers. A difference could exist between stated and observed willingness to pay for HIV prevention services. This study did not consider altruistic willingness to pay in determining the maximum amounts and proportion of people willing to pay for others to access PrEP, HIVST, and condom services. The contingent valuation approach used in this study was the bidding game. Although this is one of the valuation methods that most accurately depicts the Nigerian market iteration process, studies have highlighted gaps and challenges with this method based on its starting point bias.^15^ This study did not collect data on the socioeconomic index of the respondents, so the actual ability of the respondents was not measured. Therefore, future studies should endeavor to compute the socioeconomic status of the respondents.

## CONCLUSIONS

Considering dwindling donor and government funding for HIV prevention services, Nigeria cannot afford universal free HIV prevention services. Among those willing to pay for HIV prevention commodities, gaps still exist between the amount they are willing to pay and the current market prices of the commodities. There is a need to institutionalize a system to bridge the gap between the maximum amount the KP groups are willing to pay and the retail prices. If prices of these commodities are reduced, the willingness to pay may result in high consumption and returns on businesses in the private sector.

## Notes

**Peer Reviewed**

**First published online:** September 13, 2022.
